# Chemo-mechanical modeling of smooth muscle cell activation for the simulation of arterial walls under changing blood pressure

**DOI:** 10.1007/s10237-023-01700-x

**Published:** 2023-03-09

**Authors:** Klemens Uhlmann, Daniel Balzani

**Affiliations:** Chair of Continuum Mechanics, Ruhr-Universität Bochum, Universitätsstraße 150, 44801 Bochum, Germany

**Keywords:** Smooth muscle, Contraction, Artery, MLCK, MLCP

## Abstract

In this paper, a novel chemo-mechanical model is proposed for the description of the stretch-dependent chemical processes known as Bayliss effect and their impact on the active contraction in vascular smooth muscle. These processes are responsible for the adaptive reaction of arterial walls to changing blood pressure by which the blood vessels actively support the heart in providing sufficient blood supply for varying demands in the supplied tissues. The model is designed to describe two different stretch-dependent mechanisms observed in smooth muscle cells (SMCs): a calcium-dependent and a calcium-independent contraction. For the first one, stretch of the SMCs leads to an inlet of calcium ions which activates the myosin light chain kinase (MLCK). The increased activity of MLCK triggers the contractile units of the cells resulting in the contraction on a comparatively short time scale. For the calcium-independent contraction mechanism, stretch-dependent receptors of the cell membrane stimulate an intracellular reaction leading to an inhibition of the antagonist of MLCK, the myosin light chain phosphatase resulting in a contraction on a comparatively long time scale. An algorithmic framework for the implementation of the model in finite element programs is derived. Based thereon, it is shown that the proposed approach agrees well with experimental data. Furthermore, the individual aspects of the model are analyzed in numerical simulations of idealized arteries subject to internal pressure waves with changing intensities. The simulations show that the proposed model is able to describe the experimentally observed contraction of the artery as a reaction to increased internal pressure, which can be considered a crucial aspect of the regulatory mechanism of muscular arteries.

## Introduction

In 1902, William Maddock Bayliss observed the contraction of an artery as a result of an increase in the intravascular pressure (Bayliss 1902; Blum et al. 1999). This reaction constitutes the most important tool of arterioles and capillaries for regulating the blood flow and protecting the tissue from damage caused by overstretching. Today, it is known as the Bayliss-effect and associated with the contraction of smooth muscles cells (SMCs) in the media of the arterial wall. To analyze atherosclerotic arteries with respect to, e.g., efficiency and influence of antihypertensive drugs or plaque development and rupture, patient-specific numerical simulations are considered a promising toolbox for the improvement of clinical practice. For this purpose, an accurate calculation of the stress and strain distribution throughout the arterial wall is essential, which requires computational simulation to include all biomechanically relevant aspects, cf. e.g., Uhlmann et al. (2022). Under physiological conditions, these aspects include the passive material response of elastin and collagen (Holzapfel 2000; Balzani et al. 2006), residual stresses (Chuong and Fung 1986; Balzani et al. 2007; Ambrosi et al. 2011; Cyron and Humphrey 2017; Zahn and Balzani 2018) as well as the active material response (Yang et al. 2003b; Murtada et al. 2012; Böl et al. 2012; Stålhand et al. 2011; Yosibash and Priel 2012). While modeling each aspect has its own difficulties, here, we focus on the modeling of the active response and embrace already existing approaches for the passive material response and the residual stresses. Furthermore, we do not consider any effects in the material response which are associated with supra-physiological loading conditions. These may include microscopic damage, which can be induced in tissues, e.g., during balloon angioplasty. Then, appropriate models allowing for the mesh-independent simulation of stress-softening phenomena can be applied, cf. e.g., Balzani and Ortiz (2012) and Schmidt and Balzani (2016).

SMCs are located in all hollow organs and contribute to their biological functionality. As in other muscle types, the degree of active contraction in SMCs is mainly governed by the cytosolic free calcium concentration. An increase of the calcium concentration can be caused by a release of calcium from the sarcoplasmic reticulum (SR; an ion-storage located within the cell membrane) or from extracellular calcium influx through calcium ion-channels. Both sources can be triggered by various biological signals including, e.g., calcium waves, junctional calcium transients, calcium sparks, calcium puffs, and L-type calcium channel sparklets (Amberg and Navedo 2013; Wray 2010). While the experimental data to identify the contribution of every single mechanism is still limited, the current state of knowledge identifies the calcium influx into vascular SMCs as a primarily stretch-dependent process, cf. Cole and Welsh (2011), Johnson et al. (2009), Ji et al. (2002), Gao (2017), Pfitzer (2019), Schneider (2013) and Wizemann (2012). In addition to that, another mechanosensitive contraction mechanism has to be taken into account, which we will refer to as the calcium-independent contraction mechanism. This contraction mechanism is especially important when it comes to the contraction behavior over a longer time frame. While the increase of the cytosolic free calcium concentration is stimulating the activity of the enzyme MLCK (myosin light-chain kinase), the stretch of receptors of the cell membrane also leads to an inhibition of the antagonist of MLCK, which is MLCP (myosin light-chain phosphatase). Both, the stimulation of MLCK and the inhibition of MLCP, contribute significantly to the intensity of contraction. Hence, the goal of this paper is to derive a phenomenological approach to model the calcium-dependent and calcium-independent contraction of vascular SMCs.

One of the most common model approaches for muscle shortening was published by Hill (1938). It illustrates the mechanical behavior and heat production during muscle shortening as a system of two components, where a visco-elastic part and a contractile part are arranged in series. There, the visco-elastic component represents the mechanical behavior of the elastic tissue, while the contractile component serves as a model of the muscle. An extension of this model is formulated in Fung (1970), which adds a parallel elastic component, representing the elasticity of the muscle tissue at rest. This is also known as three-element Hill muscle model. Another base for the modeling of smooth muscles was set by the model of cross-bridge phosphorylation by Hai and Murphy (1988). Contractile units consist of myosin and actin proteins, which are responsible for the contraction of SMCs. In their model, Hai and Murphy describe the chemical dependency of myosin light-chains on the concentration of cytosolic free calcium to activate MLCK. This is considered as the only regulatory mechanism for the ability of myosin heads to perform power strokes. This chemical model is well-accepted and used in many publications from the last two decades (Murtada et al. 2010a, 2012; Yang et al. 2003a, b; Böl et al. 2012; Stålhand et al. 2011). Murtada et al. (2010a) use a phenomenological approach to develop a new material model for smooth muscle contraction. They combine the three-element Hill muscle model with the chemical model for cross-bridge phosphorylation and consider the intracellular free calcium concentration as input, which is not influenced by mechanisms of the model. In a subsequent publication (Murtada et al. 2012), the authors include a mechanical description of the overlap between myosin and actin filaments, influencing the degree of active contraction. Similar to striated muscle, they define the filament overlap in SMCs as increasing for the raise of the active contraction. With this formulation of the filament overlap, they are able to match the presented experimental data. In this context, a study by Liu et al. (2013) reported that the length of myosin filaments is actually widely varying and relatively short. Another theory about the length of myosin filaments can be found in Chitano et al. (2017). They measured the concentration of monomeric free myosin at different states of muscle stretch. It was found that the amount of monomeric myosin is decreasing in stretched SMCs. Myosin in its monomeric form has no contribution to the contraction mechanism. However, Chitano et al. (2017) suggest that monomeric myosin is consumed for polymerization, extending the myosin filaments, which they assume to be the predominant mechanism for the increase of active contraction after muscle stretch. Especially the polymerization of monomeric myosin confirms that the overlap between myosin and actin provides a considerable contraction mechanism for SMCs. Furthermore, the filament sliding during a contraction of a SMC can lead to an elongation of the overlap between both filaments. Hence, the approach to modify the magnitude of the driving stress inside of SMCs based on the overlap of the contraction filaments is convincing and works especially well from a numerical point of view to fit experimental data of the corresponding papers. More recent publications towards this contraction mechanism can be found in Haspinger et al. (2018), Murtada et al. (2017) and Stålhand and Holzapfel (2016). However, while this effect can have considerable impact on the degree of contraction, the majority of reports in biology and medicine (see, e.g., Wray 2010; Cole and Welsh 2011; Tykocki et al. 2017; Johnson et al. 2009; Ji et al. 2002; Gao 2017) support the approach to model the increase in active contraction as a process governed by the influx of calcium ions. One model for smooth muscle contraction with such an approach was published by Yang et al. (2003a). The authors included an electrochemical model part by combining a Hodgkin–Huxley-type membrane model with models of the fluid compartment. While this represents one of the most detailed approaches to include the calcium influx from the extracellular medium and the SR, it also requires a detailed amount of experimental data. In Böl et al. (2012), the calcium concentration in SMCs was defined as a time-dependent calcium function. This simple approach enabled the investigation of calcium waves moving through the artery without defining a coupling between the chemical model and the mechanics. Another promising material model for smooth muscle contraction is described in Stålhand et al. (2011). Here, the influence of the chemical state of the SMCs onto their mechanical behavior is characterized by a modified version of the model for cross-bridge phosphorylation by Hai and Murphy (1988). By introducing stretch-dependent reaction rates inside the chemical model, the amount of phosphorylated, attached cross-bridges increases for larger fiber stretch, causing a stronger active contraction. This particular model was also tested by the authors of this paper and revealed a numerical issue, where the reaction rates were able to become negative for a high stretch, which is non-physiological. In Yosibash and Priel (2012), an active material model for SMC contraction was proposed where the active stress was modeled dependent on the concentration of a vasoconstrictor and the current stretch in fiber direction. The model was fitted to experimental data from Wagner and Humphrey (2011) where endothelin-1 was the investigated vasoconstrictor. The model by Yosibash and Priel (2012) was further investigated in Gilbert et al. (2019) by coupling the mechanics with a diffusion of the vasoconstrictor. While the model showed a promising fit to experiments, it should be recognized that several hormones influence the SMC contraction of the arterial wall at the same time. A more complex version of this model could be considered to embrace the contractile mechanism of arteries in-vivo. Two other recent publications introduce mechano-electrochemical models for the contraction of SMCs in the uterus and the urinary bladder (Sharifimajd et al. 2016; Seydewitz et al. 2017). Both models consider the membrane potential as the most important activation factor for calcium influx via L-type voltage-gated calcium channels. The change of the membrane potential of SMCs in larger hollow organs can be initiated by a various number of stimuli, e.g., the nervous system or by stretch-dependent mechanisms. On the one hand, both model approaches support our own understanding of calcium influx in SMCs of the arterial wall. On the other hand, the influx of calcium into vascular SMCs is mostly mechanosensitive and not directly comparable to the influx of calcium in larger hollow organs.

For the purpose of modeling vascular smooth muscle contraction, we will focus on the calcium-dependent and calcium-independent mechanisms by extending the model of Hai and Murphy (1988) and combining it with a modified version of Murtada et al. (2012). While other possible mechanisms for the contraction of smooth muscle in the arterial wall were mentioned above, the increase of an overlap between actin and myosin as well as the influence of vasoconstrictors are not included in our approach. To describe the passive, hyperelastic material behavior of the arterial wall, we adopt the polyconvex material model of Balzani et al. (2006). The functionality of the proposed model will be presented by replicating experimental data from Johnson et al. (2009), where a middle cerebral rat artery was subjected to different levels of internal pressure while the temporal adjustment of the outer diameter was measured. There, not only the active material response was investigated, but also the mechanical reaction of the artery under the influence of Rho kinase inhibitors, which are known to be suppressing the contraction mechanism of MLCP. Since certain aspects of the artery in the experiment are unknown, we will present reasonable assumptions concerning the geometry of the arterial ring, the boundary conditions, fiber directions and the determination of the material parameters. The assumptions are related to both, own experience in the numerical work with arterial simulations and scientific references. We apply the opening angle method on the arterial ring to include circumferential residual stresses (Chuong and Fung 1986). It will be shown that the model is able to fit the experimental data accurately, especially for the purely active contraction at higher pressure values. The artery considered in the experiments is a middle cerebral artery of a rat and is thus, a muscular artery. In contrast to elastic arteries such as the aorta, muscular arteries are found further away from the heart. They are richer in SMC content, especially in the media (center layer of the artery) and they contain a high amount of collagen in the adventitia (outer layer), for more information see (Murtada and Humphrey 2018). In addition, they produce a lower amount of nitric oxide (see Leloup et al. 2015) which makes them more vulnerable when it comes to cardiovascular diseases as atherosclerosis. Especially because of the high amount of SMCs, muscular arteries are able to hold or even decrease their outer diameter after an increase of the blood pressure. Therefore, we further investigate the proposed model by applying pressure waves to an arterial ring. These simulations demonstrate the performance of the proposed model to replicate the contractile behavior of a muscular artery which restores its diameter even after a distinct increase of the blood pressure.

## Chemo-mechanical model for active vascular tissue

We focus on muscular blood vessels, such that the tissue consists of elastin, collagen, and a dominant fraction of active SMCs. For the passive response, we consider the model from Balzani et al. (2006) and combine it with reasonable assumptions about geometry and fiber orientation in an arterial ring. Most important in this section, however, is the introduction of a coupling between the chemical model of the active material with its mechanical model part. We extend the models of Hai and Murphy (1988) and Murtada et al. (2012) by a set of new stretch- and rate-dependent equations to describe the chemical reactions of the calcium-dependent and -independent contraction mechanisms.

### Continuum mechanical basis and passive response

Let $\varvec{X}$ be a material point in the reference configuration ${\mathcal {B}}$ in the three-dimensional space, and $\varvec{x}$ the material point in the current configuration ${\mathcal {S}}$. The motion of a material point $\varvec{X}$ is defined by the time-dependent map $\varvec{x}= \varphi _t(\varvec{X}) = \varphi (\varvec{X}, t)$. The deformation gradient $\varvec{F}$, which is associated with the map $\varphi$, and the right Cauchy-Green tensor $\varvec{C}$ are given by

1 $$\begin{aligned} \varvec{F}= \frac{\partial \varvec{x}}{\partial \varvec{X}} \,, \quad \varvec{C}= \varvec{F}^{\text {T}} \varvec{F}\,. \end{aligned}$$

In order to account for the passive, hyperelastic material behavior of the arterial tissue, the material model is developed based on the finite strain theory. In addition, the concept of structural tensors $\varvec{M}^{(f)}=\varvec{a}^{(f)} \otimes \varvec{a}^{(f)}$ (cf. e.g., Boehler 1987) is applied to address the anisotropy resulting from the embedded fibers. The fiber directions $\varvec{a}^{(f)}$ are regarded, which are arranged helically around the vessel wall. A weak interaction of these two fiber families is assumed, which allows for an additively decomposed formulation of the strain-energy density function $\Psi$. The isotropic energy part $\Psi _{\text {p}, \, \text {isot}}$ describes the elastin-rich matrix in which collagen fibers and SMCs are embedded. The orientation of embedded fibers in soft biological tissues can be arbitrarily complex and simulations will always require suitable simplifications. The specific orientations depend also strongly on the location of the artery in the body. Due to a lack of specific information regarding SMC orientation in the particular artery considered later, we decided to not follow oversimplified assumptions, where only one fiber direction in circumferential direction is considered. Instead, for the orientation of smooth muscle, we follow similar approaches from the literature, see, e.g. Haspinger et al. (2018), where the SMCs are mainly oriented in the direction of the mainly two collagen fiber families, cf. Horny et al. (2010). We additionally consider a qualitatively realistic distribution of fiber angle (measured between the circumferential and axial direction) along the radial direction. Though, we do not account for dispersed fibers and thus, our assumptions represent to some extent a simplification. However, in the analysis presented in Sects. 3 and 4, we focus on mostly qualitative effects in a simplified arterial segment. Therefore, not including sophisticated and thus very specialized SMC orientations or even dispersion is reasonable. Hence, the material behavior along the fiber directions is described by four transversely isotropic parts here, namely, $\Psi _{\text {p}, \, \text {ti}}^{(1)}$ and $\Psi _{\text {p}, \, \text {ti}}^{(2)}$ for the passive collagen fibers, and $\Psi _\text {a}^{(1)}$ and $\Psi _\text {a}^{(2)}$ for the active SMCs. Consequently, the additive decomposition of $\Psi$ can be written as

2 $$\begin{aligned} \Psi = \Psi _{\text {p}, \, \text {isot}} + \sum _{f=1}^2 \Psi _{\text {p}, \, \text {ti}}^{(f)} + \sum _{f=1}^2 \Psi _\text {a}^{(f)} \,. \end{aligned}$$

Note that within the two summations, different fiber orientations may be considered. For a more specific definition of the strain-energy density, a coordinate-invariant representation in terms of the principal and mixed invariants is considered with

3 $$\begin{aligned}{} & {} I_1 = \text {tr} (\varvec{C}) \,, \quad I_2 = \text {tr} [ \text {Cof} (\varvec{C})] \,,\nonumber \\{} & {} I_3 = \text {det} (\varvec{C}) \,,\quad I_{4}^{(f)} = \varvec{C}:\varvec{M}^{(f)} \,,I_{5}^{(f)} = \varvec{C}^2:\varvec{M}^{(f)} \,.\nonumber \\ \end{aligned}$$

Herein, $\text {Cof}(\bullet ) = \text {det}(\bullet )^{-\text {T}}$ denotes the cofactor. It is worth mentioning that the fifth invariant is not polyconvex on its own. However, to ensure the existence of minimizers and material stability, polyconvexity is important (Ball 1977). Hence, the alternative invariant for the transversely isotropic part, which has been introduced in Schröder and Neff (2003), is considered as

4 $$\begin{aligned} K_{3}^{(f)} = I_1 I_{4}^{(f)} - I_{5}^{(f)} \,, \end{aligned}$$

which fulfills the polyconvexity condition. For the isotropic energy part, a neo-Hookean formulation is used which excludes a dependency on $I_2$. In addition, an isotropic energy part is added to represent the nearly incompressible behavior of the arterial tissue. Deviations from the incompressible state are punished by the term $\alpha _2 ( I_3^{\alpha _3} + I_3^{-\alpha _3} - 2)$. The transversely isotropic part is formulated as a function of $K_3$ according to Balzani et al. (2006) and thus, the passive components of the material model are given by

5 $$\begin{aligned} \Psi _{\text {p}, \, \text {isot}}= & {} \alpha _1 \left( I_1 I_3^{-1/3} - 3 \right) + \alpha _2 \left( I_3^{\alpha _3} + I_3^{-\alpha _3} - 2 \right) \quad \text {and}\nonumber \\{} & {} \quad \Psi _{\text {p}, \, \text {ti}}^{(f)} = \alpha _4 \langle K_3^{(f)} - 2 \rangle ^{\alpha _5} \,, \end{aligned}$$

where the material parameters are restricted to $\alpha _1 > 0$, $\alpha _2 > 0$, $\alpha _3 > 1$, $\alpha _4 > 0$ and $\alpha _5 > 2$, and $\langle \bullet \rangle =1/2\left( \bullet + |\bullet |\right)$ define the Macaulay brackets. Once, the strain energy density is defined, the second Piola-Kirchhoff and the physical Cauchy stress tensor can be computed as

6 $$\begin{aligned} \varvec{S}= 2\partial _{\varvec{C}}\Psi \quad \text{ and }\quad \varvec{\sigma }= \frac{1}{\text {det}\varvec{F}}\varvec{F}\varvec{S}\varvec{F}^{\text {T}}. \end{aligned}$$

### Mechanical–chemical coupling for active response in SMCs

The model of cross-bridge phosphorylation and regulation of the latch state in smooth muscles by Hai and Murphy (1988) describes the influence of calcium on myosin filaments. SMCs contain a network of protein filaments which are separated by dense bodies, including contractile units. Contractile units consist of thin actin filaments and thick myosin filaments arranged in parallel to each other. Cross-bridges, also known as myosin heads, are able to build a connection between both protein filaments. The entire contraction mechanism is initiated by an influx of calcium ions into SMCs, which interact with calmodulin. Calcium–calmodulin-complexes bind to the enzyme myosin light-chain kinase (MLCK), activating the phosphorylation of regulatory light-chains of myosin. As a consequence, phosphorylated myosin heads are able to attach to actin and, subsequently, perform power strokes which results in a contraction of the cell. Attached as well as detached phosphorylated myosin heads can be dephosphorylated by the activity of the enzyme myosin light-chain phosphatase (MLCP). Note that all equations in this subsection relate to one single fiber direction and thus, we skip the index (*f*) to not overcomplicate notation here. Since the mathematical expressions in Sect. 2.3 allow for a readable notation, the indices will there be used again. In the model of Hai and Murphy, the myosin heads are classified in four functional states: (A) detached and dephosphorylated, (B) detached and phosphorylated, (C) attached and phosphorylated, and (D) attached and dephosphorylated. The transformation of myosin heads from one state into another is described by the reaction rates for phosphorylation ($k_1$ and $k_6$), dephosphorylation ($k_2$ and $k_5$), attachment ($k_3$), and detachment ($k_4$ and $k_7$). Since the attachment of myosin heads can only occur for phosphorylated myosin, there is no reaction rate for the transformation of myosin heads from state *A* to state *D*. Consequently, following the model of Hai and Murphy (1988), four ordinary differential equations describe the kinetic model of the four myosin states as the following

7 $$\begin{aligned} \begin{bmatrix} \dot{n}_{\text {A}} \\ \dot{n}_{\text {B}} \\ \dot{n}_{\text {C}}\\ \dot{n}_{\text {D}} \end{bmatrix} = \begin{bmatrix} -k_1 &{} k_2 &{} 0 &{} k_7 \\ k_1 &{} -k_2 - k_3 &{} k_4 &{} 0 \\ 0 &{} k_3 &{} -k_4 - k_5 &{} k_6 \\ 0 &{} 0 &{} k_5 &{} -k_6 - k_7 \end{bmatrix} \begin{bmatrix} n_{\text {A}} \\ n_{\text {B}} \\ n_{\text {C}}\\ n_{\text {D}} \end{bmatrix} \,, \end{aligned}$$

where $n_{\text {A}}$, $n_{\text {B}}$, $n_{\text {C}}$, and $n_{\text {D}}$ constitute the proportions of myosin heads in the respective state. As natural constraints for proportions, the following equations have to be fulfilled

8 $$\begin{aligned} n_{\text {A}} + n_{\text {B}} + n_{\text {C}} + n_{\text {D}} = 1, \quad \text{ with }\quad n_{\text {A}}, n_{\text {B}}, n_{\text {C}}, n_{\text {D}} \in [0; 1] \,. \end{aligned}$$

In the system of ordinary differential Eq. (7), the reaction rates $k_3$, $k_4$, and $k_7$ are considered as constant. The reaction rates $k_1$ and $k_6$ describe the activity of MLCK, which is calcium-dependent. As calcium-independent antagonist, the reactions rates $k_2$ and $k_5$ describe the activity of MLCP. As suggested in Murtada et al. (2012), $k_1$ and $k_6$ are described as a function of $[{\textrm{Ca}}^{2+}]$, i.e.

9 $$k_{{1/6}} = \eta \frac{{[{\text{Ca}}^{{2 + }} ]^{2} }}{{[{\text{Ca}}^{{2 + }} ]^{2} + ({\text{Ca}}_{{50}} )^{2} }},$$

where $[{\textrm{Ca}}^{2+}]$ is the concentration of intracellular calcium, ${\textrm{Ca}}_{50}$ represents the half-activation constant and $\eta$ is a parameter defining the maximally achievable value for $k_1$ and $k_6$. As stated in the introduction, the influx of calcium into vascular SMCs is a complex stretch-dependent mechanism. A sudden stretch of the cell results in a fast increase of the intracellular calcium concentration. This increased level of calcium concentration leads to a rather immediate contraction of the cell to protect the tissue from overstretching. Subsequently, the increased calcium level triggers an outflow of calcium, which reacts slower than the initial inflow. Based on this outflow, the calcium concentration eventually settles at a certain constant value as long as the loading situation of the cell remains unchanged. To model the instantaneous inflow of calcium into the cell after an increase of the stretch, we propose a stretch-dependent calcium function as

10 $$\begin{aligned}{}[{\textrm{Ca}}^{2+}](\lambda ) = {\gamma _1} \langle \lambda - \overline{\lambda }_{\text {c}}\rangle ^{2} \,, \end{aligned}$$

where $\lambda = (I_4^{(f)})^{1/2}$ is the stretch in longitudinal direction of the SMC and $\gamma _1$ is a material parameter. To describe the comparably slower outflow of calcium after a sudden stretch of the cell, an evolution equation for $\overline{\lambda }_{\text {c}}$ is proposed as the four-parameter sigmoid function

11 $$\begin{aligned}{} & {} \dot{\overline{\lambda }}_{\text {c}}(\Delta [{\textrm{Ca}}^{2+}]) = \dot{\bar{\lambda }}_{\text {c}, \, \text {min}} + \displaystyle \frac{\dot{\bar{\lambda }}_{\text {c}, \,\text {max}} - \dot{\bar{\lambda }}_{\text {c}, \, \text {min}}}{1+e^{\gamma _2 (\Delta [{\textrm{Ca}}^{2+}] - \tau _{\text {c}})}}{\quad }\nonumber \\{} & {} \text {with} \,\tau _{\text {c}} = \ln \left( \frac{\dot{\bar{\lambda }}_{\text {c}, \, \text {min}} - \dot{\bar{\lambda }}_{\text {c}, \, \text {max}}}{\dot{\bar{\lambda }}_{\text {c}, \, \text {min}}}{-} 1 \right) (-\gamma _2)^{-1} \,, \end{aligned}$$

where $\dot{\bar{\lambda }}_{\text {c}, \, \text {min}}$ and $\dot{\bar{\lambda }}_{\text {c}, \, \text {max}}$ describe the minimal and maximal change rate of $\overline{\lambda }_{\text {c}}$, respectively, and $\gamma _2$ is a material parameter. In addition, $\tau _{\text {c}}$ is set as formulated in Eq. (11) to ensure that $\dot{\overline{\lambda }}_{\text {c}} (\Delta [{\textrm{Ca}}^{2+}] = 0) = 0$, which stops $\dot{\overline{\lambda }}_{\text {c}}$ from changing whenever $\Delta [{\textrm{Ca}}^{2+}] = 0$. The evolution equation for $\overline{\lambda }_{\text {c}}$ depends on $\Delta [{\textrm{Ca}}^{2+}] = [{\textrm{Ca}}^{2+}]_{\text {tar}} - [{\textrm{Ca}}^{2+}]$, where $[{\textrm{Ca}}^{2+}]_{\text {tar}}$ is the target calcium concentration. This target calcium concentration constitutes the calcium value which is reached in a steady state at a certain stretch $\lambda$. Thus, we define the target calcium concentration as

12 $$\begin{aligned}{}[{\textrm{Ca}}^{2+}]_{\text {tar}}(\lambda ) = \gamma _3 \frac{\lambda ^{2}}{\lambda ^{2}+(\lambda _{50, \, \text {c}})^{2}} \,, \end{aligned}$$

with the material parameters $\gamma _3$ and the half-activation stretch $\lambda _{50, \, \text {c}}$. For the second contraction mechanism, which is assumed to react significantly slower, the calcium-independent and mechanosensitive inhibition of MLCP is also proposed to be described by a four-parameter sigmoid evolution equation, this time for $k_{2/5}$:

13 $$\begin{aligned} \begin{aligned} \dot{k}_{2/5}(\Delta \bar{\lambda }_{\text {p}}, k_{2/5})&= \dot{k}_{2/5, \, \text {min}} \displaystyle \left( 1-e^{-\zeta _1 k_{2/5}} \right) + \frac{\dot{k}_{2/5, \, \text {max}} - \dot{k}_{2/5, \, \text {min}}}{1+e^{\gamma _4 (\Delta \bar{\lambda }_{\text {p}} - \tau _{\text {p}})}} \\ \text {with} \quad \tau _{\text {p}}&= \ln \left( \frac{\dot{k}_{2/5, \, \text {min}} - \dot{k}_{2/5, \, \text {max}}}{\dot{k}_{2/5, \, \text {min}}} - 1 \right) (-\gamma _4)^{-1} \,, \end{aligned} \end{aligned}$$

where $\Delta \bar{\lambda }_{\text {p}} = \lambda - \bar{\lambda }_{\text {p}}$. The parameters $\dot{k}_{2/5, \, \text {min}}$ and $\dot{k}_{2/5, \, \text {max}}$ are the minimal and maximal change rate of $k_{2/5}$, respectively, and $\gamma _4$ is a material parameter. In addition, the penalty parameter $\zeta _1$ in the constraint term $e^{-\zeta _1 k_{2/5}}$ of Eq. (13) ensures that $k_{2/5}$ never becomes negative, which would be non-physiological. Additionally, the equation for $\dot{k}_{2/5}$ should fulfill $\dot{k}_{2/5}(\Delta \bar{\lambda }_{\text {p}} = 0) = 0$ as long as the constraint term is zero. Therefore, the rearrangement of this equation sets the parameter $\tau _{\text {p}}$ as described in Eq. (13). Similar as in the model for the calcium-dependent contraction mechanism, $\overline{\lambda }_{\text {p}}$ is introduced here as a new stretch quantity to regulate the adaptation of $k_{2/5}$ over time after a change of the stretch $\lambda$. For this purpose, the evolution equation for $\overline{\lambda }_{\text {p}}$ is defined as

14 $$\begin{aligned} \begin{aligned} \dot{\bar{\lambda }}_{\text {p}}(\Delta k_{2/5}, \Delta \bar{\lambda }_{\text {p}})&= \dot{\bar{\lambda }}_{\text {p}, \, \text {min}} + \displaystyle \frac{\dot{\bar{\lambda }}_{\text {p}, \, \text {max}} - \dot{\bar{\lambda }}_{\text {p}, \, \text {min}}}{1+e^{\gamma _5 (\Delta k_{2/5} - \tau _{\text {k}})}} - \dot{\bar{\lambda }}_{\text {p}, \, \text {max}} \, e^{-\zeta _2 ( \Delta \bar{\lambda }_{\text {p}} - \Delta \bar{\lambda }_{\text {p}, \, \text {min}})} \\ \text {with} \quad \tau _{\text {k}}&= \ln \left( \frac{\dot{\bar{\lambda }}_{\text {p}, \, \text {min}} - \dot{\bar{\lambda }}_{\text {p}, \, \text {max}}}{\dot{\bar{\lambda }}_{\text {p}, \, \text {min}}} - 1 \right) (-\gamma _5)^{-1} \,, \end{aligned} \end{aligned}$$

where $\dot{\bar{\lambda }}_{\text {p}, \, \text {min}}$ and $\dot{\bar{\lambda }}_{\text {p}, \, \text {max}}$ are the minimal and maximal change rate of $\overline{\lambda }_{\text {p}}$, respectively, and $\gamma _5$ is a material parameter. Here, the penalty parameter $\zeta _2$ ensures that $\Delta \bar{\lambda }_{\text {p}}$ is always larger than $\Delta \bar{\lambda }_{\text {p}, \, \text {min}}$, which leads to a slow relaxation of the calcium-independent contraction mechanism. To guarantee that $\dot{\bar{\lambda }}_{\text {p}}(\Delta k_{2/5} = 0) = 0$ while the constraint function $\dot{\bar{\lambda }}_{\text {p}, \, \text {max}} \, e^{-\zeta _2 ( \Delta \bar{\lambda }_{\text {p}} - \Delta \bar{\lambda }_{\text {p}, \, \text {min}})}$ is zero, the parameter $\tau _{\text {k}}$ is set as described in Eq. (14). While the constraint function is dependent on $\Delta \bar{\lambda }_{\text {p}}$, which has been introduced above, the four-parameter sigmoid function of Eq. (14) is only dependent on $\Delta k_{2/5} = k_{2/5, \, \text {tar}} - k_{2/5}$, where $k_{2/5, \, \text {tar}}$ is considered as the target value which is reached after the stretch of a cell stayed for a long time at a rather constant value. This target value for the activity of MLCP is defined as dependent on $\lambda$:

15 $$\begin{aligned} k_{2/5, \, \text {tar}} = \gamma _6 \left( 1 - \frac{\lambda }{\lambda _{50, \, \text {p}} + \lambda } \right) \,, \end{aligned}$$

with the material parameter $$\gamma _6$$ and the half-activation stretch $$\lambda _{50, \, \text 
{p}}$$.

### Chemical–mechanical coupling for active response in SMCs

The active material model of the present work is based on the approach in Murtada et al. (2012). However, while (Murtada et al. 2012) consider the three-element Hill muscle model (Hill 1938) as foundation to additively split the fiber strain into an active and an elastic part, we found this approach not accurate in arbitrary situations. In fact, significant elastic strains may be reached, which could lead to non-physical, negative values of the active strain. Hence, we suggest a multiplicative split of the fiber stretch $\lambda ^{(f)}$ such as

16 $$\begin{aligned} \lambda ^{(f)} = \lambda ^{(f)}_{\text {a}} \lambda ^{(f)}_{\text {e}} \,, \end{aligned}$$

where $\lambda ^{(f)}_{\text {e}}$ describes the elastic stretch of the cell in fiber direction (*f*) and $\lambda ^{(f)}_{\text {a}}$ is the active stretch which is governed by the contraction of the SMCs. This approach is in line with classical multiplicative splits of the deformation gradient into elastic and inelastic parts, which goes back to, e.g., Lee (1969). We keep the idea of the three-element Hill muscle model where the passive and the active material component of the artery are acting as parallel. This means in particular, that the coupling between passive and active material is assumed as weak, which supports the additive decomposition of the strain-energy function as formulated in Eq. (2). While the passive material has already been described, here, we focus on the coupling of chemically-induced smooth muscle contraction and the elastic elongation of SMCs. The elastic energy stored inside of the SMCs is considered dependent on the elastic stretch $\lambda _\text {e}^{(f)}$. Additionally, only attached myosin cross-bridges can contribute to the elastic material response. Hence, the active strain-energy function in fiber direction (*f*) is defined as

17 $$\begin{aligned} \Psi _\text {a}^{(f)} = \frac{\mu _a}{2} \left( n^{(f)}_\text {C} + n^{(f)}_\text {D} \right) \left( \lambda _{e}^{(f)} - 1 \right) ^2 \,, \end{aligned}$$

where $\mu _\text {a}$ constitutes a stiffness constant, and $n_\text {C}$ and $n_\text {D}$ are the proportions of myosin heads in the attached states $\text {C}$ and $\text {D}$. The elastic stretch can be obtained by rearranging Eq. (16) to $\lambda _\text {e}^{(f)} = \frac{\lambda ^{(f)}}{\lambda _\text {a}^{(f)}}$. The contraction of the SMCs is governed by the cycling of the myosin cross-bridges in state $\text {C}$ and results in a sliding between actin and moysin filaments, which is expressed through $\lambda _\text {a}^{(f)}$. The contraction behavior of a muscle can be investigated by quick-release experiments, where the shortening velocity of the tissue is measured for a certain afterload. The shortening velocity can be set into relation to the afterload by using Hill’s equation for tetanized muscle contraction (Katz 1939), which is defined as

18 $$\begin{aligned} (F+a)(v+b) = (F_0+a)b \,, \end{aligned}$$

where *F* is the isotonic afterload, $F_0$ is the isometric force at which the quick-release is performed, *v* is the muscle shortening velocity, and *a* and *b* are fitting parameters. Considering the muscle shortening velocity related to the speed, by which the active strain changes, we can substitute *v* with $\dot{\lambda }_\text {a}^{(f)}$, the first time derivative of the active strain $\lambda _\text {a}^{(f)}$. Furthermore, the afterload *F* can be replaced by the active stress $P_\text {a}^{(f)}=\partial \psi _\text {a}^{(f)} / \partial \lambda ^{(f)}$ which is obtained after isotonic contraction, and $F_0$ can be expressed by a driving stress $P^{(f)}_\text {c}$, which is related to the cycling cross-bridges. Hence, we obtain a similar hyperbolic function as in Eq. (18) which can be rewritten as

19 $$\begin{aligned} \dot{\lambda }_\text {a}^{(f)} = \beta _1 \frac{P_\text {a}^{(f)} - P^{(f)}_\text {c}}{P_\text {a}^{(f)} + \beta _2} \,, \end{aligned}$$

where $\beta _1$ and $\beta _2$ are material parameters. The active stress $P_\text {a}^{(f)}$ can directly be obtained from the active part of the strain-energy function $\Psi _\text {a}^{(f)}$ as

20 $$\begin{aligned} P_\text {a}^{(f)} = \mu _\text {a} \left( n^{(f)}_\text {C} + n^{(f)}_\text {D} \right) \left( \lambda _{e}^{(f)} - 1 \right) \,. \end{aligned}$$

The driving stress $P^{(f)}_\text {c}$ depends on the chemical state of the myosin in SMCs, namely, the proportion of myosin heads $n_\text {C}$ which are able to perform power strokes. It is defined as

21 $$\begin{aligned} P^{(f)}_\text {c} = \kappa \, n^{(f)}_{\text {C}}\,, \end{aligned}$$

where $\kappa$ is the maximal achievable driving stress.

### Numerical implementation

**Fig. 1:**
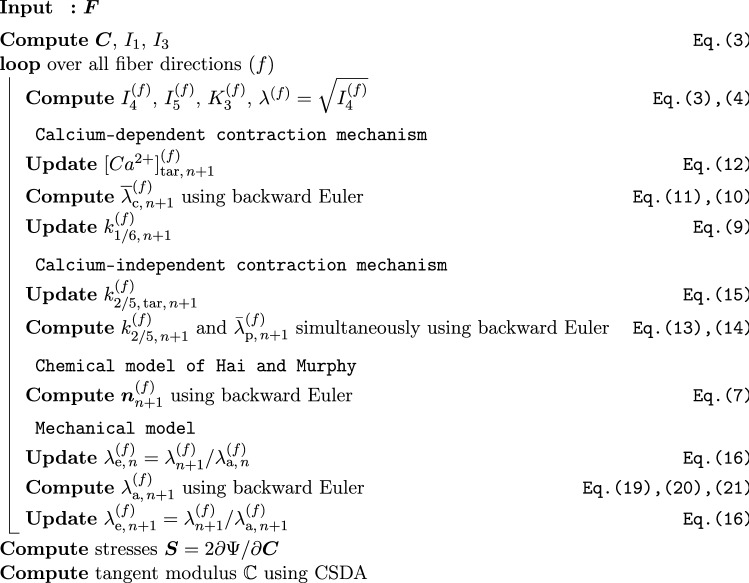
Algorithm to calculate the second Piola–Kirchhoff stresses and the tangent moduli at a material point from the proposed material model. All quantities have to be computed for all fiber directions, separately

The proposed model has been implemented into *FEAP* (Finite Element Analysis Program) using a multilevel Newton scheme. For this purpose, the tangent moduli, i.e. the derivatives of the stresses with respect to strains, which are needed for the global Newton–Raphson scheme, were computed by using complex-step derivative approximation (CSDA) following (Tanaka et al. 2014). All evolution equations are numerically integrated by applying the backward Euler integration scheme (Butcher 2003). To solve the backward Euler integration scheme iteratively (Ortega and Rheinboldt 1970), a local Newton iteration was implemented for Eqs. (11), (13), (14), and (19). Since Eqs. (13), (14) are coupled in the sense that both are dependent on $k^{(f)}_{2/5}$ and $\overline{\lambda }^{(f)}_{\text {p}}$, they are solved simultaneously. Furthermore, updates for the stretch $\lambda ^{(f)}$ in fiber direction (*f*), which are coming from the Newton iteration of the global finite element problem, are considered as an update of the elastic part of the stretch $\lambda ^{(f)}_e$. Hence, $\lambda ^{(f)}_a$ is only changing by solving Eq. (19). The algorithmic implementation scheme for calculating the stresses and the tangent moduli at a certain Gauss point is illustrated in Fig. 1.

## Parameter adjustment and comparison to experimental data

**Fig. 2:**
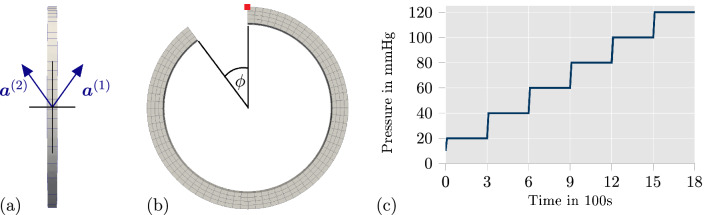
Visualization of the mesh with **a** the fiber angles in the plane of longitudinal and circumferential direction, **b** the opening angle to apply residual stresses, and **c** a diagram of the pressure profile, which was used in experimental data and applied for the parameter adjustment of the proposed model. The red square in **b** marks the node which is used in Fig. 5 to plot the evolution of mechanical and chemical quantities over time

In this section, the parameters of the proposed model are adjusted to experimental data from Johnson et al. (2009) (see Fig. 4a in the original publication). There, the contraction of a segment of a rat’s middle cerebral artery was investigated in Krebs solution by applying a sequence of intravascular pressure with increasing pressure values and, meanwhile, measuring the change of the outer diameter over time. Every pressure level was held over a time span of 300 s. The used pressure protocol is illustrated in Fig. 2c. The pressure was applied to the same artery three times, where the surrounding Krebs solution was varied to obtain different mechanical responses:

1. Normal Krebs solution - fully active contraction of a healthy artery,
2. Krebs solution including 1 $\upmu$M of Rho kinase inhibitor Y27632—contraction of an artery with suppressed calcium-independent mechanism,
3. Krebs solution with zero calcium concentration - no contraction; passive material response of the artery.

All three scenarios were considered when adjusting the material parameters. In order to enable a suitable comparison of the model response acting at the material point level and the experimental data which was obtained from a structural problem, we replicated the experimental setup by defining a corresponding boundary value problem in *FEAP*. A mesh for an arterial ring was created with one element in longitudinal direction, four elements in radial direction and 72 elements in circumferential direction, which results in 288 quadratic, 20-node brick elements in total. In the experimental setup, the cerebral artery is fixated on the cannulas of the arteriograph with nylon threads and then, no additional prescribed axial stretches are applied. This setup prevents the entire artery from axial movement, and axial strains close to zero should be expected throughout the specimen. Therefore, we decided to set the displacements in axial direction for the arterial ring in our simulation to zero. The diameters of the unloaded artery were not provided in Johnson et al. (2009). However, a wall thickness of 20 $\upmu$m and a ratio of 0.2 between wall thickness and lumen is reasonable for such an artery (see Table 1 in Gannon et al. 2008). Therefore, the inner and outer radius of the unloaded and stress-free state of the arterial ring was selected as 92 and 112 $\upmu$m, respectively. To apply reasonable fiber directions, a constant gradient for the fiber direction over the wall thickness was defined, based on data from Schriefl et al. (2012), see also the numerical results in Fausten et al. (2016). Note that human elastic arteries were used to measure fiber directions in Schriefl et al. (2012). Although fiber directions in a middle cerebral rat artery may vary from these measurements, experience during setting up the optimization problem showed that the influence of the fiber directions on the qualitative response of the model can be expected small, although some optimized parameters may be different. However, we tried to be as realistic as possible and thus, we did not include a constant fiber angle over the wall thickness. Considering an even more complicated fiber orientation would give the optimization problem more flexibility and thus, even better agreement with experiments should be expected. The fibers lie in the plane of longitudinal and circumferential direction (see Fig. 2a). The angle between fiber and circumferential direction starts with $10^{\circ }$ at the inner side and increases linearly to $40^{\circ }$ at the outer side of the arterial wall. The collagen fibers and the SMCs were assumed to be aligned in the same directions. In addition, residual stresses were considered by applying the opening angle approach. The corresponding opening angle $\phi$ of the arterial ring (see Fig. 2b) was included as a fitting parameter to match the experimental data of the passive material response. To fit the parameters to the experimental data, an optimization was implemented into *python* by utilizing the library *mystic*. *Mystic* offers a mixture of evolution strategy and gradient methods. For every set of parameters which was newly generated from *mystic*, *FEAP* is called inside the *python* script to solve the described boundary value problem with the new parameter set. Thanks to the parallelization of *mystic*, up to 40 children parameter sets were analyzed at the same time. The objective function

22 $$\begin{aligned} z = \displaystyle \sqrt{\sum _{k=1}^{n_{\text {data}}} \left( \frac{d_{\text {exp}, \, k}-d_{\text {sim}, \, k}}{d_{\text {exp}, \, k}} \right) ^2 } \end{aligned}$$

was defined to measure the differences between the model response and the experiment. Herein, $d_{\text {exp}, \, k}$ is the measured, outer diameter from the experimental data at the time $t_k$, and $d_{\text {sim}, \, k}$ is the outer diameter from the simulation at the same time $t_k$. The optimization of the material parameters, i.e. minimization of the objective function, was executed in two sequential steps: First, the five passive material parameters and the opening angle $\phi$ of the arterial ring were fitted by running an optimization which only accounted for the experimental data from the scenario three, i.e. where the passive response is tested. The resulting values for those six parameters are listed in Table 1.

**Table 1:** Optimized passive parameters and opening angle

Parameter	$\alpha _1$ (kPa)	$\alpha _2$ (kPa)	$\alpha _3$	$\alpha _4$ (kPa)	$\alpha _5$	$\phi$
Value	11.52507	151.73775	2.75662	1.27631	3.08798	$38.923^{\circ }$

In a second step, the experimental scenarios one and two were simulated in *FEAP* sequentially to enable the optimization of the parameters of the calcium-dependent and -independent contraction mechanism simultaneously. This means, in particular, that data from experiments and simulations of the scenario one and two were evaluated together in the objective function given in Eq. (22). In both scenarios, time-dependent contractile mechanisms have to be considered. To reproduce the initial contractile state of the arterial wall, we apply an intravascular pressure of 10 mmHg over a time of 600 s before the pressure profile starts. Since the agent Y27632 is suppressing the calcium-independent contraction mechanism, we assume that the reaction rates $k_{2/5}$ are constant in scenario two. Hence, we introduce an additional fitting parameter $k_{2/5, \text {const}}$, which equals the reaction rates $k_{2/5}$ in scenario two. In addition to all fitting parameters of the proposed model, reasonable starting values have to be set for the time dependent quantities. Such starting values are labeled as $\bullet _{\text {start}}$. The initial value for the fraction of myosin heads in state *A* was set to $n_{\text {A}, \, \text {start}}=1$. According to Eq. (8), the starting values for $n_{\text {B}, \, \text {start}}$, $n_{\text {C}, \, \text {start}}$ and $n_{\text {D}, \, \text {start}}$ were set to 0. Table 2 lists all additional chemical parameters which were set prior to the optimization. Note that these parameters were preselected based on experience gathered from previous optimization runs to speed up the optimization procedure.

**Table 2:** Chemical material parameters, manually adjusted/set prior to optimization

$k_3$	$k_4$	$k_7$	$Ca_{50}$	$\gamma _2$	$\gamma _3$	$\lambda _{50, \, \text {c}}$	$\bar{\lambda }_{\text {c}, \, \text {start}}$	$\lambda _{\text {a}, \, \text {start}}$
$0.134\,\text {s}^{-1}$	$0.00166\,\text {s}^{-1}$	$0.000066\,\text {s}^{-1}$	$0.4\,\upmu \text {M}$	$50\,\upmu \text {M}^{-1}$	$0.9\,\upmu \text {M}$	1.2	1.0	1.0

$\gamma _4$	$\zeta _1$	$\gamma _5$	$\zeta _2$	$\Delta \bar{\lambda }_{\text {p}, \, \text {min}}$	$\gamma _6$	$\lambda _{50, \, \text {p}}$	$\bar{\lambda }_{\text {p}, \, \text {start}}$	$\lambda _{\text {e}, \, \text {start}}$
200	$100\,\text {s}$	$50\,\text {s}$	1000	$-\,0.00001$	$1.5\,\text {s}^{-1}$	1.0	1.0	1.0

Aside from $\beta _2 = 26.68$ kPa (see Murtada et al. 2012), all mechanical parameters were part of the optimization. The constant reaction rate for $k_{2/5}$ in scenario 2 was optimized to be $k_{2/5, \text {const}} = 0.892345\,\text {s}^{-1}$. All other chemical and mechanical parameters, which were part of the optimization, are listed in Table 3.

**Table 3:** Active mechanical and chemical parameters, optimized

$\eta$	$\gamma _1$	$\dot{\bar{\lambda }}_{\text {c}, \, \text {max}}$	$\dot{\bar{\lambda }}_{\text {c}, \, \text {min}}$	$\dot{k}_{2/5, \, \text {max}}$	$\dot{k}_{2/5, \, \text {min}}$
$0.1624\,\text {s}^{-1}$	$0.5131\,\upmu \text {M}$	$0.0443\,\text {s}^{-1}$	$-\,0.0443\text {s}^{-1}$	$0.0009735\,\text {s}^{-2}$	$-\,0.0010694\text {s}^{-2}$

$\dot{\bar{\lambda }}_{\text {p}, \, \text {max}}$	$\dot{\bar{\lambda }}_{\text {p}, \, \text {min}}$	$\mu _\text {a}$	$\kappa$	$\beta _1$	$k_{2/5, \, \text {start}}$
$0.0000699\,\text {s}^{-1}$	$-\,0.0002323\,\text {s}^{-1}$	$11.857\,\text {kPa}$	$148.262\,\text {kPa}$	$0.001006\,\text {s}^{-1}$	$1.82758\,\text {s}^{-1}$

**Fig. 3:**
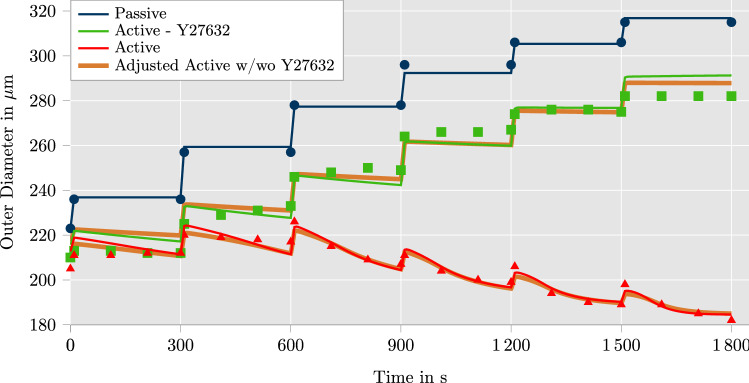
Comparison of model response with experimental data from Johnson et al. (2009) for three different setups: passive response, active response under influence of 1 $\upmu$m Rho kinase inhibitor Y27632 and fully active response. The Rho kinase inhibitor Y27632 is assumed to block the calcium-independent contraction mechanism ($k_{2/5}$ is const.). The results of the model nearly correspond with the experimental data. An adjusted variation of the parameter optimization is illustrated in orange. In this case, the parameter optimization incorporates a decrease of calcium concentration and calcium flow rate while Y27632 influences the artery. The corresponding results are almost identical to the original computation

**Fig. 4:**
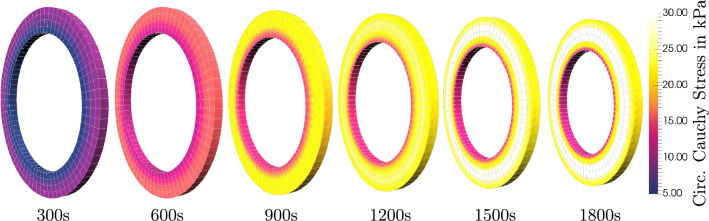
Comparison of circumferential Cauchy stresses of the fully active material response of Fig. 3 at different times of the pressure profile (see Fig. 2c); Stresses increase up to 30 kPa for an intravascular pressure of 120 mmHg; stress gradients over the wall thickness are significantly reduced at all times due to application of residual stresses

The comparison between the selected data points of the experiments, which were manually extracted from the original paper, and the simulation results with optimized parameter sets is illustrated in Fig. 3. In addition to that, Fig. 4 shows 3D contour plots of the circumferential Cauchy stress of the active material response at the end of each pressure level. Stresses up to 30 kPa are reached for an intravascular pressure of 120 mmHg which are found in the middle of the wall in radial direction. Stress gradients over the wall thickness are clearly visible for all times, but they are significantly reduced due to the application of residual stresses. Note that instead of the simple opening angle strategy followed here, more sophisticated models for residual stresses based on growth may be applied following the constraint mixture theory (Humphrey and Rajagopal 2002) or growth-related deformation gradients (Zahn and Balzani 2018). Such models would enable more homogeneous transmural distributions of stresses, which is in line with the homeostasis concept cf. Cyron and Humphrey (2014). However, as can be seen in the diagram of Fig. 3, the model shows an accurate representation of the experimental data.

So far, our parameter adjustment was based on the assumption that MLCP can indeed be deactivated by the agent Y27632 to separate the response resulting from MLCK. However, in the paper by Jackson and Boerman (2017), it is shown that Y27632 also influences the level of intracellular calcium concentration and thus, the inflow rate of calcium ions into the SMCs which will have an effect on MLCK. Unfortunately, in Johnson et al. (2009), the potentially modified calcium concentration has not been measured as part of the experiment. In order to investigate if our model is able to represent the experiment even for the scenario when the calcium concentration is changed by the agent, we performed an additional parameter optimization. Motivated by data from Jackson and Boerman (2017), we made an estimation for the modified calcium concentration based on the text above Fig. 8a in Jackson and Boerman (2017), where the concentration of the intracellular calcium was found to be lowered to 67.3%. Since the concentration of Y27632 injected there was 10 times the concentration injected in the experiments considered here (experiments from Johnson et al. 2009), we used the diagram in Fig.  7 of Jackson and Boerman (2017) to estimate the lowering of the calcium concentration to approximately 75%. Additionally, a clear lowering of the number and frequency of calcium waves was shown in Fig. 8a of Jackson and Boerman (2017). However, due to a lack of specific data, we also assumed a reduction of the calcium flow into the cells to 75%. Therefore, for our analysis of the scenario where the agent is applied, we decreased the parameter values of $\gamma _1$ and $\gamma _3$ to 75% of their values corresponding to the fully active response. The reduction of $\gamma _1$ results in a direct decrease of the calcium flow into the cell. Additionally, lowering $\gamma _3$ decreases the target value of the intracellular calcium concentration at a certain stretch. The results are indicated by “adjusted active w/wo Y27632” in Fig. 3 and the associated parameters are listed in Table 4 in the Appendix. Results show, that the proposed model is able to represent the experiments just as well even if the effect of agent-based, altered calcium concentrations is taken into account. Admittedly, the modified scenario is based on some estimations where findings from Jackson and Boerman (2017) were transferred to the data in Johnson et al. (2009) and thus, the considered scenario may not be perfectly realistic. However, the reasonable agreement for different scenarios shows already that the mechanisms included in the model appear to be appropriate. Experiments show that a significant contraction of the artery is obtained even when the internal pressure is increased. This effect is essential for the functional principle of blood flow regulation in many muscular arteries, which not only stiffen but also contract upon increased blood pressure to assist the heart. The accurate representation of this response governs the significance of computational simulations. Since our model agrees well with the experiments even for this specific response, it appears to be a reasonable approach to describe the loading-dependent adaptation of the active response of vascular tissue. Competitive approaches, e.g., Murtada et al. (2012), Haspinger et al. (2018), include the stretch-dependency directly in the mechanical part of the active stress model by considering a specific microscopic assumption regarding a stretch dependent change of myosin-actin overlap length. For small muscular arteries, this change of overlap length can be considered small and thus, this cannot explain the significant effects observed in the experiments in Johnson et al. (2009). This may explain, that, to the best of the authors knowledge, existing models have so far not been shown to be able to represent vascular contraction upon increased internal pressure as observed in the experiments in Fig. 3. On the other hand, MLCP and MLCK are known mechanisms which also modify active stress intensity upon external stretch, although through more chemical effects. Therefore, our model represents an approach which is close to the biochemical processes since it incorporates the stretch-dependency directly in the chemical part of the model which is in line with observations known as the Bayliss effect. This does apparently not only allow for a suitable representation of the experiments, it also allows a more direct and biophysically motivated incorporation of further effects on the active response, e.g., induced by antihypertensive drugs.

## Numerical analysis of proposed model

In this section, the proposed model is analyzed further to show the general capabilities of the model and to illustrate why the incorporation of the Bayliss effect is important. For this purpose, the material point level as well as a structural simulation will be considered.

### Investigation of the contraction at a material point

**Fig. 5:**
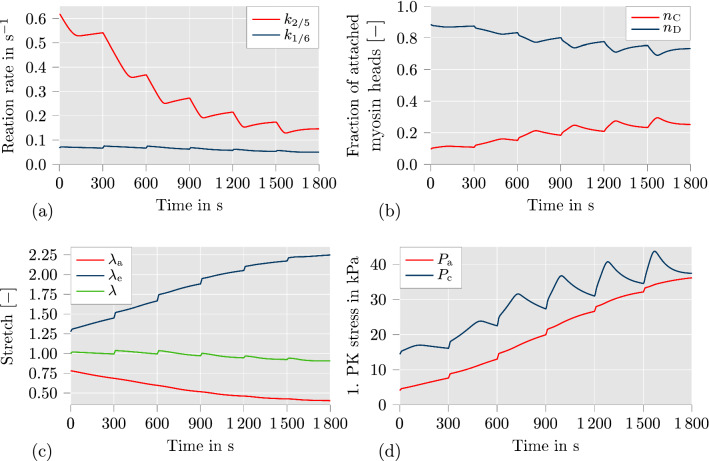
Plots of mechanical and chemical quantities over time for the simulation setup with fully active contraction (described in Sect. 3) at the marked node in Fig. 2b: **a** reaction rates $k_{1/6}$ and $k_{2/5}$; **b** fraction of attached myosin heads $n_{\text {C}}$ and $n_{\text {D}}$; **c** active stretch $\lambda _{\text {a}}$, elastic stretch $\lambda _{\text {e}}$ and total stretch $\lambda$ in fiber direction; **d** active stress $P_\text {a}$ and driving stress $P_\text {c}$

To further investigate the details of the contractile mechanism of the proposed model, we provide diagrams showing the evolution of important quantities over time in Fig. 5. The data is plotted for one fiber direction of the marked node in Fig. 2b, which is located at the outer diameter of the arterial ring, using the simulation setup for the fully active material model as described in Sect. 3.

Because of the symmetry of geometry and fiber angles, the plotted quantities are equal for both fiber directions. In the first diagram, the evolution of the reaction rates $k_{1/6}$ and $k_{2/5}$ are shown, which are identified with the activity of MLCK and MLCP, respectively. It can be clearly seen that the downregulation of $k_{2/5}$ is the predominant contraction mechanism here. This can be explained by the decision to fit the first and second experimental scenario simultaneously, where $k_{2/5}$ is constant in the second scenario. The difference in diameter between passive material response and active material response without calcium-independent contraction mechanism is only about 20 $\upmu$m over the entire time span (see Fig. 3). Hence, the calcium-dependent contraction mechanism is fitted to produce a degree of contraction which fits this small decrease of the diameter. As a consequence, the calcium-independent contraction mechanism was optimized to be much stronger to capture the contractile behavior of the fully activated artery. Based on the temporal progress of $k_{1/6}$ and $k_{2/5}$, the fraction of attached myosin heads changes as illustrated in Fig. 5b. The fraction of attached and phosphorylated myosin heads $n_{\text {C}}$ is directly proportional to the driving stress $P_\text {c}$ (see Fig. 5d). At the end of the pressure profile, $n_{\text {C}}$ reaches a value of roughly 0.25 which leads to $25\%$ of the maximally achievable driving stress. Figure 5c shows the evolution of the elastic stretch $\lambda _{\text {e}}$, the active stretch $\lambda _{\text {a}}$ and the total stretch $\lambda$. The increase of the elastic stretch constitutes another crucial mechanism in the proposed model to accomplish a high degree of active contraction of the arterial wall. It can be clearly seen that $\lambda _{\text {e}}$ reaches a value as high as 2.25. In an additive split, this would have led to a negative active stretch $\lambda _{\text {a}}$, which would probably be considered non-physical. Additionally, it can be seen that the active stretch $\lambda _{\text {a}}$ decreases smoothly over the entire duration. Only $\lambda _{\text {e}}$ and $\lambda$ show visible steps at the time points whenever the intravascular pressure is increased. This behavior is caused by the implementation choice in which $\lambda _{\text {e}}$ is updated for the current time step before the active stretch $\lambda _{\text {a}}$ is computed (see Fig. 1). Finally, Fig. 5d shows the evolution of the active stress $P_\text {a}$ and the driving stress $P_\text {c}$. As long as $P_\text {c}$ is larger than $P_\text {a}$, the contraction of the artery is triggered based on the definition of the active stretch $\lambda _{\text {a}}$ in Eq. (19). It can be seen that at the end of the last three pressure steps, $P_\text {a}$ nearly equals $P_\text {c}$, which leads to a deceleration of the contraction. As soon as $P_\text {a}$ is larger than $P_\text {c}$, a slow relaxation of the arterial wall would be initiated. Overall, it can be seen that there are three contraction mechanisms in the proposed model: The calcium-dependent contraction mechanism which controls $k_{1/6}$, the calcium-independent contraction mechanism which controls $k_{2/5}$, and the contraction via active stretch $\lambda _{\text {a}}$. All of them have to work conjointly to produce the degree of contraction which is visible in the experimental data.

### Application of pressure waves in an artery

**Fig. 6:**
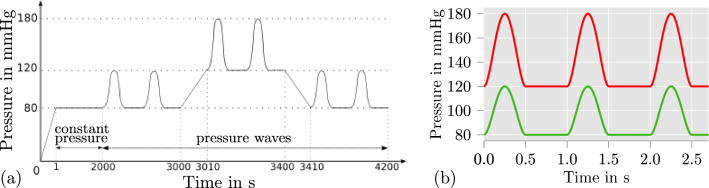
**a** Applied load protocol to arterial ring (see results in Fig. 7); Note that in time regions where pressure waves are applied (time > 2000 s) a number of one pressure wave per second is considered; **b** Considered intravascular pressure waves: 120/80 mmHg (green), 180/120 mmHg (red)

To investigate the proposed model on its suitability for describing the contribution of SMC contraction to the blood flow regulation, we define simple intravascular pressure waves which are meant to mimic idealized heart-beat-like variations in pressure. In general, the diameter of smaller arteries decreases when the blood pressure increases (Blum et al. 1999) which is due to the active contraction of SMCs. Because of this functionality, these smaller arteries are called resistance arteries. The contractile behavior of resistance arteries increases the resistance of the arteries towards the blood flow and ensures that the blood during high body activity is actually reaching the muscles which are in need of additional oxygen-rich blood. The geometry, mesh and Dirichlet boundary conditions, which were described in Sect. 3, are kept for the investigations here. For the analysis of patient-specific arteries, approaches as, e.g., proposed in Balzani et al. (2012) can be considered, but here, we are more interested in an idealized scenario to enable a decent qualitative investigation of the general features of the individual model components. The load protocol for the entire simulation is illustrated in Fig. 6a. Firstly, the arterial ring is set under a constant pressure of 80 mmHg which is held over a time span of 2000 s. Afterwards, intravascular pressure waves for a blood pressure of 120/80 mmHg are applied. As shown in Fig. 6a, the pressure waves are increased to 180/120 mmHg at 3000 s and decreased back to 120/80 mmHg at 3400 s. The values of the pressure waves are with 120/80 mmHg and 180/120 mmHg in the physiological area of blood pressures for rats (see, e. g., Table 2 in Wang et al. 2013). The considered functions for both pressure waves are depicted in Fig. 6b. Note that the illustrated waves represent an idealized scenario which can however be considered characteristic, although not realistic. However, this simple approach is sufficient to qualitatively investigate the proposed model in a load scenario which is closer to the in vivo situation.

**Fig. 7:**
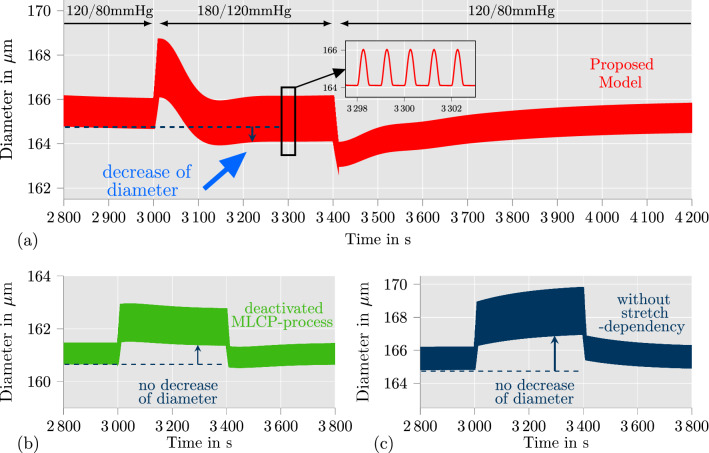
Arterial ring under intravascular pressure waves (see Fig 6) with different models: **a** proposed model; **b** proposed model without calcium-independent contraction mechanism ($k_{2/5}$ is constant); **c** proposed model without stretch-dependencies of the chemical model ($k_{2/5}$ and $k_{1/6}$ is constant). After an increase of the pressure from 120/80 mmHg to 180/120 mmHg, the application of the proposed model leads to a decrease of the arterial diameter (see **a** between 3100 and 3400 s) which is significantly visible for the diastolic blood pressure. The reduced models in **b** and **c** cannot achieve this contractile behavior

To investigate the results of the simulation, we plot the outer diameter of the arterial ring over time from 2800 to 4200 s in Fig. 7a.

First, it can be recognized that the diameter of the arterial ring is at all times smaller than the diameters depicted in Fig. 3 in which the pressure steps from Fig. 2c was applied. This can be explained by the difference of the load scenarios. The pressure waves are frequently triggering the stretch-dependency of the calcium-dependent and -independent contraction mechanism. This is not occurring during a constant intravascular pressure. Hence, the contraction of the artery is even higher for a blood pressure of 120/80 mmHg than for the constant pressure of 120 mmHg in Fig. 3. In addition, it can be seen that after roughly 900 s of pressure waves with a blood pressure of 120/80 mmHg (at 2900 s), the arterial ring develops a repetitive material response towards the pressure waves. Such a repetitive material response can also be observed at roughly 3200 s in the diagram (for a blood pressure of 180/120 mmHg) and at roughly 4100 s (for the second time span of a blood pressure of 120/80 mmHg). Especially the similarity of the repetitive material response for the first and second load period at a blood pressure of 120/80 mmHg should be noted. Based thereon, we can state that the proposed model for SMC contraction is able to provide a stable, repeatable material response for more complex load scenarios, such as pressure waves on an arterial ring considered here. In addition, it can be observed that the maximal diameter of the arterial ring is nearly equal during the repetitive material response for a blood pressure of 180/120 mmHg (see Fig. 7a, 3200–3400 s) compared to the repetitive material response at a blood pressure of 120/80 mmHg (see Fig. 7a, 2900–3000 s). This indicates that the model is, in fact, able to replicate the behavior of arteries which contributes to the blood flow regulation. To underline the necessity of the calcium-dependent and -independent contraction mechanism to cover the feature of blood flow regulation, we also investigate variations of the proposed model for the same boundary value problem. In the first variation, we exclude the calcium-independent contraction mechanism by setting $k_{2/5}$ to a constant value. To achieve similar diameter values, we adjusted the reaction rate to be $k_{2/5} = 0.03$ s${}^-1$. In the second variation, we excluded all stretch-dependencies from the chemical model by additionally keeping $k_{1/6}$ constant, which was done by setting $[Ca^{2+}] = 0.25\upmu \text {M}$. The adjustments to set $k_{1/6}$ and $k_{2/5}$ to constant values results in a quantitatively comparable model as in (Murtada et al. 2012). It can be seen from Fig. 7b and c that neither of the adjusted models can achieve qualitatively comparable contractions when the blood pressure is increased to 180/120 mmHg. Furthermore, while the model without calcium-independent contraction mechanism in Fig. 7b is still lowering the diameter directly after the blood pressure was increased (see between 3000 and 3100 s), the model without any chemically related stretch-dependency in Fig. 7c acts more like a viscoelastic than an active material. It can be concluded, that a stretch-dependency of the chemical model is essential for reliable simulations of in-vivo arteries under changing blood pressure. This could be achieved here by taking the calcium-dependent and -independent contraction mechanism of the arterial wall into account.

## Conclusion and outlook

Major novelty in the proposed formulation has been the incorporation of the calcium-dependent and -independent contraction mechanism to describe the Bayliss-effect. The mechanical model part was based on Murtada et al. (2012); the chemical model on Hai and Murphy (1988). According to the calcium-dependent and -independent contraction mechanism, a set of new equations was introduced which describe reaction rates of the chemical model as time- and stretch-dependent. The proposed model was implemented into *FEAP* and an optimization for fitting experimental data of Johnson et al. (2009) was built in *python* by utilizing the library *mystic*. Based on the optimized parameter set, the proposed model achieved a good fit to the experimental data. It could be shown that all contraction mechanisms of the model are involved to accomplish the correct contraction over time. In addition, simulation results of arterial rings under time-dependent intravascular pressure were shown. These were designed to mimick changes in blood pressure, as e.g. appearing during heart beats, as well as changes of blood pressure levels resulting, e.g., from a change of body activity. In total, three model variations were analyzed including the proposed model and two modifications. While the first model reduction excluded only the calcium-independent contraction mechanism, both stretch-dependent mechanisms of the chemical model were deactivated in the second reduction. As it turned out, the proposed model showed a realistic contractile behavior which could be identified as the Bayliss-effect, whereas the reduced models were not able to describe this. The simulation results suggested that the calcium-dependent and -independent contraction mechanism are essential in a reliable model for vascular smooth muscle whenever changes in blood pressure are to be analyzed. Consequently, our numerical investigations of the proposed model show the following benefits compared to competitive approaches:

- effective description of the contraction of muscular arteries as a result of increased internal pressure
- direct chemo-mechanical representation of the contractile mechanism in terms of a stretch-dependent modification of MLCK and MLCP
- advantageous basis for the inclusion of further chemo-mechanical effects, e.g., related to the influence of antihypertensive drugs such as calcium channel blockers or angiotensin II receptor blockers which locally affect MLCK and MLCP in vascular SMCs

When it comes to the correct prediction of deformations and stresses in patient-specific arteries, several additional aspects would be necessary to be included into the model. Firstly, the concentration of hormones is not constant over the day, which generally varies the ability of SMCs to contract. A similar modification of the strain-energy density function as in Yosibash and Priel (2012) could be considered to account for the effect of vasoconstrictors on the contraction of SMCs. Furthermore, if patient-specific arteries are to be investigated, more realistic residual stress distributions and fiber directions will be important to improve the accuracy of the simulations. In addition to that, simulations of patient-specific arteries are specifically interesting from a clinical point of view, when they are performed for diseased arteries. This means not only that degenerated tissue may be required to be considered, also the impact of antihypertensive drugs on the arterial wall has to be included into the model, which are widely prescribed to patients with cardiovascular diseases. For this purpose, the proposed model offers several access points for extensions. Calcium-ion channel blockers can be involved into the model by defining single parameters of the MLCK activity as dependent on the concentration of the corresponding agent. Other antihypertensives as angiotensin-II-receptor blockers or agents from the sartan family can be considered by manipulating the intensity of the stretch-dependency of both, the calcium-dependent and -independent contraction mechanism. With the suggested modifications, an actual toolbox for computational simulations of patient-specific arteries to optimize medical treatments could be realized.

## Data Availability

Data may be made available on request.

## Appendix: Optimized parameters for the adjusted, active case

**Table 4:** Active mechanical and chemical parameters with suppressed concentration and flow rate of calcium when SMCs are under the influence of Y27632 (see results in orange in Fig. 3), optimized

$\eta$	$\gamma _1$	$\dot{\bar{\lambda }}_{\text {c}, \, \text {max}}$	$\dot{\bar{\lambda }}_{\text {c}, \, \text {min}}$	$\dot{k}_{2/5, \, \text {max}}$	$\dot{k}_{2/5, \, \text {min}}$
$0.1905\,\text {s}^{-1}$	$0.5833\,\upmu \text {M}$	$0.05\,\text {s}^{-1}$	$-\,0.05\,\text {s}^{-1}$	$0.0018301\,\text {s}^{-2}$	$-\,0.0010865\,\text {s}^{-2}$

$\dot{\bar{\lambda }}_{\text {p}, \, \text {max}}$	$\dot{\bar{\lambda }}_{\text {p}, \, \text {min}}$	$\upmu _\text {a}$	$\kappa$	$\beta _1$	$k_{2/5, \, \text {start}}$
$0.00003983\,\text {s}^{-1}$	$-\,0.00035\,\text {s}^{-1}$	$24.153\,\text {kPa}$	$160.799\,\text {kPa}$	$0.000525\,\text {s}^{-1}$	$1.30029\,\text {s}^{-1}$
